# Violence against health care workers in a crisis context: a mixed cross-sectional study in Eastern Democratic Republic of Congo

**DOI:** 10.1186/s13031-023-00541-w

**Published:** 2023-10-03

**Authors:** Samuel Lwamushi Makali, Jean Corneille Lembebu, Raïssa Boroto, Christian Chiribagula Zalinga, Daniella Bugugu, Emmanuel Lurhangire, Bigirinama Rosine, Christine Chimanuka, Pacifique Mwene-Batu, Christian Molima, Jessica Ramirez Mendoza, Giovanfrancesco Ferrari, Sonja Merten, Ghislain Bisimwa

**Affiliations:** 1 Ecole Régionale de Santé Publique, Université Catholique de Bukavu, Bukavu, Democratic Republic of the Congo; 2 Hôpital Provincial Général de Référence de Bukavu, Bukavu, Democratic Republic of the Congo; 3 Centre de Connaissance en Santé en RDCongo, Kinshasa, Democratic Republic of the Congo; 4 Ecole de Santé Publique, Université Libre de Bruxelles, Bruxelles, Belgium; 5 International Committee of the Red Cross, Geneva, Switzerland; 6 Department of Epidemiology and Public Health, Swiss Tropical and Public Health Institute, University of Basel, Basel, Switzerland; 7 Faculté de Médecine, Université de Kaziba, Kaziba, Democratic Republic of the Congo

**Keywords:** Violences, Health care workers, Health crisis, Eastern DRC

## Abstract

**Background:**

Health Care Workers (HCWs) in conflict zones face high levels of violence while also playing a crucial role in assisting the population in distress. For more than two decades, the eastern provinces of the Democratic Republic of the Congo (DRC), have been wracked by conflict. This study aims to describe the state of violence against HCWs and the potential prevention mechanisms in eastern DRC.

**Methods:**

In North and South Kivu, between February 5 and 21, 2021, we conducted a mixed cross-sectional convergent study in health facilities (health centers and hospitals). An anonymized self-administered questionnaire was sent to HCWs about their experience of violence in the 12 months prior to the study. In-depth individual interviews with HCWs, present on the day of the investigation, were also done to explore their experience of violence. A descriptive analysis of the quantitative data and a thematic analysis of the qualitative data was carried out.

**Results:**

Of a total of 590 participants, 276 (45.9%) reported having experienced violence in the 12 months before the study. In North Kivu, aggressors were more frequently the patients (43.7% vs. 26.5%) and armed group members (14.3% vs. 7.9%) than in South Kivu. Most respondents (93.5%) reported verbal aggression (insults, intimidation, death threats). Other forms of physical aggression including with bare hands (11.2%), firearm (1.81%), and stabbing (4.7%). Only nearly one-tenth of the attacks were officially reported, and among those reported a higher proportion of sanctions was observed in South Kivu (8.5%) than in North Kivu (2.4%). The mechanisms proposed to prevent violence against HCWs were community initiatives and actions to strength the health system.

**Conclusions:**

In Eastern DRC, HCWs face multiple and severe forms of aggression from a variety of individuals. The effects of such levels violence on HCWs and the communities they served could be devastating on the already pressured health system. Policy framework that defines the roles and responsibilities for the protection of HCWs and for the development and implementation of preparedness measures such as training on management of violence are possible solutions to this problem.

## Background

The issue of violence against health care workers (HCWs) has become an increasingly important problem across the world in recent years, further worsened with the arrival of epidemics and pandemics that have terrified the whole humanity to date [1–3]. The World Health Organization (WHO) defines aggression as “the intentional use of physical force or power, threatened or actual, against oneself, another person, or against a group or community that results in, or has a high likelihood of resulting in, injury, death, psychological harm, maldevelopment or deprivation» [4, 5]. The WHO has also developed models to help countries implement strategies to prevent and mitigate violence in the health sector [6].

Although the protection and respect of HCWs is crucial for the Geneva Conventions of 1949 and their additional protocols [7, 8], HCWs continue to come under aggression around the world. During armed conflicts, HCWs are often obliged to offer the minimum care without safeguarding the medical ethics to preserve their lives [9, 10]. However, efforts have already been made in some countries to develop multidisciplinary response against HCW’s aggression or protect them in armed conflict [11–14].

There has been very little research done on this issue in DRC. A study conducted in Katanga, in pacific context, showed that 80% of HCWs were subjected to some form of aggression [15]. Aside from this work, aggression against HCWs in the DRC is reported in isolation. In 2021, 127 incidents of aggression against HCWs by armed groups were reported in the six provinces of the Eastern DRC [16]. Of these six provinces, North and South Kivu have been suffering from more than 25 years of violent instability with a high number of deaths linked to armed conflicts [17]. Insecurity and instability began with the arrival of more than two million displaced people fleeing the 1994 genocide in Rwanda, followed by the various wars in DRC (1996, 1998, 2004) and the proliferation of non-state armed groups that still operate in some areas today [18]. Furthermore the region experienced its first Ebola epidemic [19] in a context of mistrust between the population and the relief workers [20] before plunging into the Covid-19 pandemic [21, 22]. There are several problems integrated in the response to Ebola in North Kivu, including the mistrust of the population, scepticism of the disease’s existence and attacks on medical facilities [23, 24]. In the last few years, aggression on HCWs have been reported in these two provinces, ranging from minor physical assaults to the destruction of health infrastructure and in some cases, death [16, 25].

The objective of this study was to investigate the current state of aggression against HCWs (frequency, profile of aggressors, possible causes and types of aggression) and to identify the factors contributing to aggression against HCWs while determining the mechanisms in place that may contribute to the prevention of such aggression in a comparative and descriptive way across both provinces. This was done in light of the complex instability and the occurrence of epidemic diseases in these two provinces.

## Methods

### Study settings

This study was carried out in the two eastern DRC provinces, North and South Kivu. The health system of the country is organized into three levels: the central level representing the decision-making and normative framework at the level of the capital, the secondary regulatory level represented by the provincial health divisions (PHD) and the operational level, the health zone (HZ), directed by the central office of the health zone (COZ). The HZ is further subdivided into three levels of patient care which are the primary care provided by the health centers (HC), the secondary care provided by the general referral hospitals (GRH), and the tertiary care provided by the teaching hospitals. Both provinces have been experiencing armed conflict and have been affected by all waves of the Covid-19 pandemic and the deadliest Ebola epidemic (10th epidemic) [26]. More than 100 armed groups are thought to operate in the DRC’s eastern region [27]. This study considered only the secondary and operational levels including general referral hospitals (GRH), secondary hospitals and Health Centres (HC) in rural and urban areas.

### Study design and period

This is a mixed cross-sectional convergent study [28] which entailed the simultaneous collection of both quantitative and qualitative data. The triangulation of these two approaches allowed to ensure the methodological consistency from both quantitative results and qualitative findings [29]. The study was carried out from the 5th to the 22nd of February 2021.

### Selection of health zones and study participants

The selection of the study areas was made by convenience, considering accessibility during the survey period.

Overall, the study was carried out in two Provincial Health Divisions (PHD), three Central office of the zone (COZ), 24 Health Facilities (HF) including three Health Centers (HC), 14 hospitals and two teaching hospitals, in the two provinces. The persons who participated in the quantitative survey were HCWs who were present on the day of the visit from the investigators in their respective hospitals and institutions; and who provided consent to participate in the interview.

Participants in the qualitative surveys were identified in different categories of HCW from the selected HZs. The study team interviewed a total of 29 participants, including four health authorities, 12 members of general hospital coordination and administrative staff, and 13 HCWs.

### Data collection

The study team collected quantitative data using an anonymous self-administered questionnaire to capture HCWs’ experience of aggression. The questionnaire included questions related to the socio-demographic data (age, gender, profession, workplace) of the participants, whether they experienced aggression or witnessed aggression towards their colleagues, the kind of aggression they experienced, the aggressor’s profile, as well the mechanisms for reporting the aggression and whether prevention strategies were in place. Even though the questionnaire was anonymous, 98% of respondents answered despite their workload facing the epidemics.

To collect qualitative data, the research team developed an interview guide in French. The interview guide contained questions relating to the respondent’s experience of aggression and the mechanisms in place to reduce such aggression. Four interviewers (two in South Kivu and two in North Kivu) with a master’s degree in public health and prior training in qualitative methods conducted the interviews.

Data collection was facilitated by a preparatory phase consisting of preparing the respondents in advance through the provincial health divisions of the two provinces. The self-administered questionnaires were distributed to the participants in the facilities and were collected during working hours and a few hours later.

### Data analysis

The quantitative data collected were encoded in Epi Info 7 and analyzed with Stata 16. Descriptive statistics (frequency and proportion) were calculated for the categorical variables. The chi-square test with a significance threshold < 0.05 was used to compare the proportions of the variables across the two study settings (North Kivu and South Kivu).

The qualitative data were recorded in the form of audio files with Sony dictaphones in French and Swahili. These audios were then transcribed into a Word file only in French. A deductive thematic analysis [30] was conducted, based on a thematic framework comprised of two main themes inspired by the study research objective: (1) Respondents’ experience with aggression against HCW, description of the types of violence, the perpetrators, the possible causes of aggression); (2) the mechanisms in place to prevent such violence (i.e. the strategies in place to prevent and mitigate aggressions). Verbatims from the interviewees’ own words were chosen as illustrative quotes. To maintain the anonymity of the interviewees, the quotes were cited by the respondent’s number according to the order of interviews, without specifying the province or health zone from which they came from.

## Results

### Characteristics of the participants and aggression frequency

A total of 590 out of 602 HCWs (98%) were able to complete the self-administered questionnaire (Table 1). Almost half of the HCWs (45.8%) had experienced violence in the 12 months prior to our study.

**Table 1:** General characteristics of participants in the two provinces

Variables	Total [N(%)]	South-Kivu[ N(%)]	North-Kivu [N(%)]	p-value
**Aggression in last 12 months (590)**				0.067
Yes	276 (45.85)	172 (42.79)	104 (52.00)	
No	314 (52.16)	223 (55.47)	91 (45.50)	
No response	12 (1.99)	7 (1.74)	5 (2.50)	
**Age range (n = 579)**				0.883
< 35 years	198 (34.20)	137 (34.86)	61 (32.80)	
35–50 years	318 (54.92)	214 (54.45)	104 (55.91)	
> 50 years	63 (10.88)	42 (10.69)	21 (11.29)	
**Sex (n = 397)**				**0.015**
Female	150 (37.78)	91 (33.70)	59 (46.46)	
Male	247 (62.22)	179 (66.30)	68 (53.54)	
**Type of health structure (n = 499)**				**< 0.001**
Primary	68 (13.63)	34 (9.42)	34 (24.64)	
Secondary	415 (83.17)	324 (89.75)	91 (65.94)	
Tertiary	16 (3.21)	3 (0.83)	13 (9.42)	
**Profession (n = 550)**				0.368
Doctors	140 (25.45)	88 (23.28)	52 (30.23)	
Dentist/ Physiotherapist/ Pharmacist	87 (15.82)	63 (16.67)	24 (13.95)	
Nurse/Laboratory technician	259 (47.09)	182 (48.15)	77 (44.77)	
Administration / Security / Others	64 (11.64)	45 (11.90)	19 (11.05)	

Most respondents to the self-administered questionnaires were men (66.3% in South Kivu and 53.5% in North Kivu), and the age group most represented was between 35 and 50 years old (54,9%). Nurse and laboratory technician were the most represented (47.09) followed by medical doctors (25,5%). Other respondents also administrative and security members (11.6%) and other practitioners (dentist, physiotherapist, pharmacist) (15.8%).

### Profile of perpetrators and types of aggression

Perpetrators of violence included patients (32.9%), their relatives (58.4%), members of armed groups who may (11.5%) or may not (10.3%) have relationship with the patients and, other members of the community (23.1%) (Table 2). Regarding the patient him/her-self acting as the aggressor, a significant difference was observed between the two provinces, with a higher proportion of patients violently aggressing HCWs in North Kivu (43.7%) than in South Kivu (26.5%) (p = 0.003). Furthermore, attacks committed by a member of an armed group were cited more frequently in North Kivu (14.3%) than in South Kivu (7.9%), with no statistical difference in proportions (p = 0.105). Other aggressors included community members (23.2%), and relatives who employed an armed group to attack HCWs (11.5%).

**Table 2:** Profile of aggressors and types of assaults on health workers who experienced violence

Variables	Total [N(%)]	South-Kivu [N(%)]	North-Kivu [N(%)]	p-value
**Profile of the aggressor in last 12 months**				
Patient (n= 273)				**0.003**
Yes	90 (32.97)	45 (26.47)	45 (43.69)	
Non	183 (67.03)	125 (73.53)	58 (57.31)	
Family member of the patient (n = 274)				0.566
Yes	160 (58.39)	97(57.06)	63(60.3)	
Non	114 (41.61)	73(42.94)	41(39.42)	
Community member (n = 272)				0.111
Yes	63 (23.16)	34 (20.00)	29 (28.43)	
Non	209 (76.84)	136 (80.00)	73 (71.57)	
Member of an armed group (n = 261)				0.105
Yes	27 (10.34)	13 (7.98)	14 (14.29)	
Non	234 (89.66)	150 (92.02)	84 (85.71)	
Family member attached to an armed group (n = 253)				0.852
Yes	29 (11.46)	18 (11.18)	11 (11.96)	
Non	224 (88.54)	143 (88.82)	81 (88.04)	
**Types of aggression in last 12 months**				
Verbal aggression (n = 276)				0.378
Yes	258 (93.47)	162 (94.19)	96 (91.31)	
Non	18 (6.53)	10 (5.81)	8 (7.69)	
Physical assault barehanded (n = 276)				0.362
Yes	31 (11.23)	17 (9.88)	14 (13.46)	
Non	245 (88.77)	155 (90.12)	90 (86.54)	
Stabbing (n = 276)				**< 0.001**
Yes	13 (4.71)	2 (1.16)	11 (10.58)	
Non	263 (95.29)	170 (98.84)	93 (89.42)	
Assault with firearm (n = 276)				0.914
Yes	5 (1.81)	3 (1.74)	2 (1.92)	
Non	271 (98.19)	169 (98.26)	102 (98.08)	
Other assaults (n = 274)				0.271
Yes	2 (0.73)	2 (1.17)	0 (0.00)	
Non	272 (99.27)	169 (98.83)	103 (100.00)	

Verbal assaults were the most frequently reported type of violence (93.5%). Physical assaults with bare hands (11.2%), stabbings (4.7%), assaults with firearms (1.8%), and other types (n = 0.73) were also reported in both provinces. Stabbings were reported at a significant higher rate in North Kivu (10.6%) than in South Kivu (1.2%).

### Reporting of aggression and proportion of punished aggressors

**Fig. 1:**
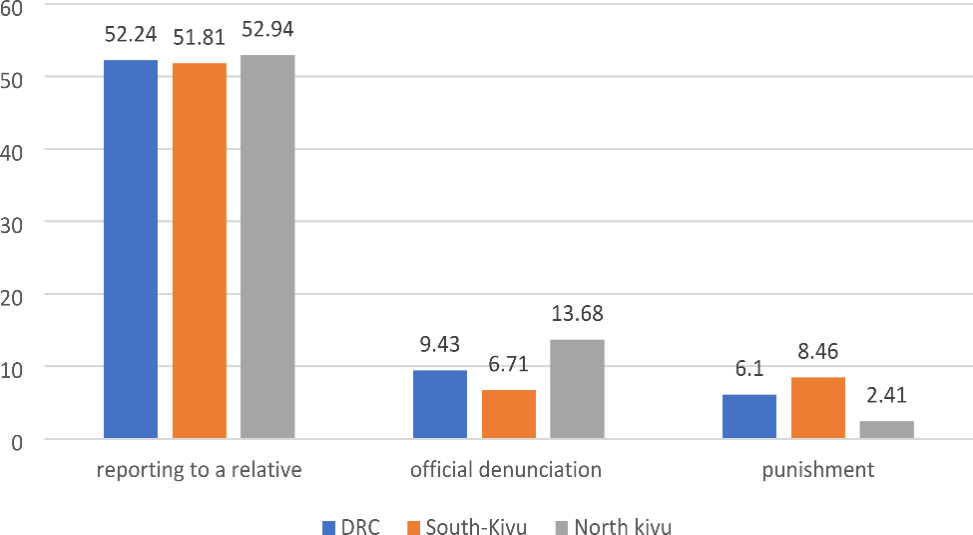
Reporting the aggression and proportion(%) of legal action (punishment) taken for aggressors

Half of the respondents reported the aggression experience to a relative in both provinces. Almost a tenth of the aggression (9.4%) were officially reported by the health system or the police, of which only 6% for which legal action was taken, with a higher proportion of legal proceedings in South Kivu (8.5%) than in North Kivu (2.4%) (Fig. 1).

### Proposed prevention strategies against violence

Four types of actions were proposed as strategies to prevent violence by the respondents: (1) political actions to curb armed groups (24.3%), (2) actions to improve health system (capacity building of HCWs, strengthening of collaborative relationships, amicable conflict resolution, improving the management of human resources and compensation) (55.3%), (3) improve the socio-economic well-being of the community (44.6%), and (4) actions to support professional organisations (doctors’ and nurses’ associations) in their activities (0.7%) (Table 3).

**Table 3:** Strategies to prevent violence against health workers as proposed by study participants

Variables	Total [N(%)]	South-Kivu [N(%)]	North-Kivu [N(%)]	p-value
**Political actions (n = 530)**				0.466
Yes	129 (24.34)	86 (23.43)	43 (26.38)	
No	401 (75.66)	281 (76.57)	120 (73.62)	
**Health system (n = 528)**				0.573
Yes	292 (55.30)	200 (54.50)	92 (57.14)	
No	236 (44.70)	167 (45.50)	69 (42.86)	
**Community actions (n = 527)**				0.538
Yes	235 (44.59)	166 (45.48)	69 (42.59)	
No	292 (55.41)	199 (54.52)	93 (57.41)	
**Professional corporations (n = 527)**				0.803
Yes	4 (0.76)	3 (0.82)	1 (0.62)	
No	523 (99.24)	362 (99.18)	161 (99.38)	

### Perceptions of health workers on their experience of aggression

With the qualitative data analysis we equally explored (a) the respondents’ experience with aggressions, and (b) the solutions they proposed.

### Respondents’ experience with aggressions

In most cases patients themselves were the source of violence. Furthermore, the aggressors included accompanying members of the patient such as family members or close friends. It was often reported, close friends would ignite the anger of the entire community to reach the health facility and threaten the HCWs. However, the study also revealed that HCWs were attacked by armed groups. Further, aggression was also instigated among HCWs due to hierarchy resentment and sometimes conflict of interest in the workplace (e.g., belonging to a particular tribe) and escalated to be one of the roots of aggressions by the community.

*« … But for another [person] it was the patient him/her-self who had to attack the care provider always in the same context not having accepted the diagnosis of Covid-19 that had to be communicated … » (Respondent 8)*.

*« … these are really threats of the community kind but much more at the level of the nursing staff often it is linked to conflicts of interest …. when, above all, you are not a native, so the natives want at all costs that you can really leave their environment, either their hospital or their health center or the central office of the zone…» (Respondent 10)*.

Aggression against HCWs occurred during the sudden onset of deadly epidemic diseases (Ebola and Covid-19). The primary cause of aggression was the community’s lack of acceptance, knowledge, and disinformation about the diseases. Anyone involved in the fight against these diseases was part of a conspiracy of the international community to kill the population as highlighted by the following quote:

*« … the titular nurse who was murdered because of this Ebola epidemic situation; she was blamed for what?… eating “dirty” money if I can translate literally. That she was making money on the back of sick people… » (Respondent 2)*.

The qualitative data also show that the worsening of the patient’s condition or the patient’s death leads to the aggression against the HCWs by the family members, as the family members believe the patient received inadequate care due to negligence or mismanagement of care by the HCWs.

*« … unfortunately, this patient had died, and it was the staff team that had pointed fingers where to say that no…, it is so-and-so…, it is because he had not done this, and the family members verbally assaulted the person there… » (Respondent 11)*.

Another reason for aggression towards the administrative staff was in relation to the fees for health services. Patients and their relatives felt at times they were overcharged for the services received.

*« … The verbal aggression first of all towards me personally, it is related to the billing… you bill a patient and the patient does not agree to pay these expenses and at a certain moment at the health center, the hospital blocks him/her, he/she is going to try to escape … while escaping he is caught, he starts the fight and also inflammatory remarks… » (Respondent 4)*.

A low level of schooling and lack of education among the population on health-related issues and the different diseases and health services were also reported as causes of aggression during this period.

« *… Ah it’s education, you must educate the population. That’s what I can say. Educate the population through awareness. Because especially… I tell you that, the people who attacked us were especially during the Ebola period… » (Respondent 5)*.

Among the verbal forms of aggression in health facilities and in the community, intimidation, insults, and death threats were the most frequently types of aggression reported. Some people working in the Ebola response and the HCWs raising awareness and providing information sessions about the reality of the Ebola virus disease have been marginalised and given mocking nicknames.

*« … They think that we are accomplices, and they say that they have already given us the money to do this. That’s why I told you that they even burned the house of the pastor where he carried the wash basin, they burned down the houses… » (Respondent 9)*.

Sadly, severe forms of physical aggression were also observed during the study and included the killing of HCWs while performing their duties. Other HCWs were victims of direct physical aggression including in the forms of barehanded blow, stone throwing, and the use of firearms. As a result, HCWs suffered severe physical consequences including injuries and fractures.

*« … You must also know that there were even deaths here, attacked by armed groups, of health care workers who were on their mission. They were shot (mentioning a place), they died like that … There were two deaths that day after the attack. Two deaths by armed groups… Several times during especially this past Ebola response here, our staff were molested, our vehicles were stoned. We even missed being stoned on the ground … » (Respondent 12)*.

Furthermore, beyond the direct aggression HCWs were also victims of indirect aggression such as destruction of property and receiving death threatening leaflets.

*« … then I had received flyers at home, they came to drop it off saying that I would be kidnapped … » (Respondent 1)*.

### Mechanisms in place to reduce aggression

Respondents described some measures already in place aiming at preventing aggression against HCWs. At the community level for example, to address the lack of knowledge of Ebola and Covid-19 epidemics, awareness sessions were organized through community dialogue with the help of political and administrative authorities. Another measure mentioned and established in various HZs was psychosocial care for patients.

*« … …. We were obliged to involve the political and administrative authorities, such as the chief of the district, who helped us to raise awareness among the population through community dialogues organised in different villages…. » (Respondent 7)*.

At the level of health structure, some hospitals have invested in security services to ensure the security of the health facilities and to prevent violence from happening within the facility enclosure. Furthermore, administrative procedures and standards have been put in place by the different heads of services and include disciplinary measures for any misbehaviour to ensure a certain degree of conviviality.

*« … we try to make them aware of the fact that they should always keep professional secrecy and behave like a community, the conviviality between the team must be fused together but… there are also disciplinary measures which are there… » (Respondent 10)*.

*« … To limit that, well… at least for our structure we have the gate first. And then members of a security company help us block… when one comes to physically attack … » (Respondent 12)*.

## Discussion

The purpose of this study was to determine the patterns of violence against HCWs and to describe their experience of violence in the eastern North and South Kivu provinces of DRC affected by armed conflict and as well as during the recent Ebola and Covid-19 pandemics [31, 32].

### The frequency of attacks on health care workers

In this study, 46% of our respondents reported having experienced aggression in the exercise of their profession at least once in the 12 months prior to the interviews which is consistent with previously documented studies including in Nigeria (40%) [33]. However, a higher prevalence of violence against HCWs has been reported in Cote D’Ivoire (78%) [34], Egypt (59.7%), Saudi Arabia (67.4%), Palestine (80.4%) [35], in Iraq (85%) [36], and and even in the province of Katanga in DRC (80.1%) [15] which, moreover, was not experiencing armed conflict nor an Ebola epidemic during the study period. The prevalence variations could be attributed to methodological differences including the choice of respondents (doctors, nurses or all HCWs including administrative staff), definitions of violence employed, the type of care or health facilities investigated (huge variations have been documented in teaching hospitals and psychiatric facilities for example). Furthermore, due to the differences between geographical scope (urban vs. rural) of the studies and the socio-political context in the different regions is difficult to make comparisons. The only study previously conducted in DRC in 2015, included over 2,000 HCWs from medical to receptionists, technicians, and administrative staff from over 400 health facilities located in a province not affected by armed conflict. As such, the large proportion of the incidents were verbal in nature (almost 60%) [15].

### Types and causes of violence against health workers

Regarding the many types of violence recorded, this study found that verbal violence (insults, intimidation, threat of murder) was the most prevalent (93,5%) and that physical violence (killing, hitting and wounds including those inflicted by stones) and other types had a very low prevalence. Indeed, a WHO report published in 2019, reported some attacks in the DRC against Ebola treatment sites resulting in the death of some staff [37]. These results are also in line with those of Al Nazi et al. in Saudi Arabia (83% for verbal abuse and 5% for physical abuse) [38], et Rafeea et al. in Bahrain (78% for verbal abuse and 11% for physical abuse) [39]. However, a study done in Katanga in the DRC reported a lower prevalence of verbal aggression (57%) than in the present study [15]. This disparity could be explained by the fact that Katanga is a stable security zone. Other studies, such as that conducted in Egypt by Abdellah et al., showed similar findings but with lower estimates of the prevalence of non-physical violence [40].

In North Kivu [41], there was a higher incidence of stabbing [42] than in South Kivu, which aligns with findings from a study about conflict dynamics during the Ebola outbreak by Kraemer et al. Indeed, the researchers found that conflict dynamics are much higher in North Kivu and Ituri Province compared to other provinces in the DRC [43]. Furthermore, in the study areas of North Kivu there are multiple armed groups (whose members are part of the local communities) and that commonly use knives and machetes. Aggression using firearms were not widely reported but are significant because of their devastating consequences. A similar trend was reported in Ethiopia, Malawi and Kenya, regarding emotional abuse and sexual harassment among co-workers [44–47] and in Pakistan regarding some incidents of firearm violence [48]. Beyond verbal and physical violence, HCWs reported receiving death threats via messages on small papers.

The main factors contributing to violence against HCWs were attributed to deeply rooted mistrust over the health response emanating from rumours about the two epidemics and confirmed by previous studies [49, 50], as well as a perception of negligence by HCWs and dissatisfaction with patient’s care. These may be the consequence of the low level of education among the population and their lack understanding of the disease [23]. Indeed, Mueller et al. reported an increase in violence against HCWs during the Ebola outbreak in North Kivu [20] that can be explained by the perception of Ebola as a conspiracy not only to raise money but also to exterminate a part of the population [51]. Cai et al. in China reported that the perpetrators’ perception of professional misconduct after the death of a loved one was the main cause of the various forms of aggression towards HCWs [52]. The same is true when the perpetrator was the patient him/her-self, acts for aggression were attributed to dissatisfaction with the care received which could be due to insufficient personnel or neglect [38, 53]. It should be pointed out that improving the communication between HCWs and patients or family members would be a good solution to limit the majority of violence in health care services [54].

### The profile of aggressors of HCWs

At 58.4%, most of the perpetrators were family members. They not only assaulted the caregivers, but they were able to bring and entice other members of the community to commit aggression.

These findings support the observations of a systematic review on aggression against HCWs in emergency departments [55], as well as other studies conducted in Palestine [35], in Turkey [56] and Saudi Arabia [53] where the majority of the perpetrators were relatives. This could be explained by the fact that the patient’s relatives frequently arrive in large numbers to inquire about the patient’s condition and calls for the need for health facility policies to restrict the number of accompanying members. Furthermore, relatives have ongoing concerns throughout the patient’s hospitalization that the HCWs may not be able to address causing major frustrations and their inability to accept the worsening of the patient’s condition or death often exacerbates into violence. This study reveals that a small proportion of the aggression reported by HCWs was attributed to members of armed groups primarily in North Kivu. Aggression by armed groups could have more long-term devastating effects than those caused by high-frequent-low-intensity events of aggression (i.e., verbal aggression committed by relatives), but the consequences have to our knowledge not been the subject of research. It is worth mentioning however, that HCWs might feel compel to not disclose the perpetrator of such aggression and in fact almost half of them prefer not to disclose the information. In many circumstances, identifying the armed-group affiliation could be very difficult particularly when there is lack of clarity on whether an individual is acting on their own accord or on behalf of an armed group. A global review shows that only 5% of the literature available on violence against healthcare was conducted in contexts of armed-conflict [57]. The ICRC estimates that a large proportion of violence against health care in conflict affected contexts is underreported [58] .

### Declaration aggression, mechanisms of prevention of aggression and prosecution of perpetrators

Less than one tenth of the cases of violence in the present study (9.43%) were officially reported, and only 6% of those cases were prosecuted. Similar low levels of reporting were described by Al Anazi et al. in Saudi Arabia (16,4%) [38] and Al Bashtazy et al. in Jordan (16,6%) [59] described. However, when when compared to other studies conducted in Palestine (40%) and Turkey (35%) [60] our results show that the level of reporting in DRC is very poor. This difference might be linked to methodological differences (the definition of officially reported in our study excludes when aggression was reported to a relative) and above all because in the DRC there are no domestic legislation and regulations to protect HCWs against aggression. Although we did not explore the reasons for lack of reporting; other reasons explaining the low prevalence of reporting have been described elsewhere and include: fear of negative consequences such as revenge, fear of losing one’s job, the perception that reporting is useless when not supported by management or by a response to the victim, the absence of an adequate system for managing violence against HCWs, and the normalization of aggression in the workplace [15, 59, 61, 62].

Some of the aggression prevention mechanisms proposed by the respondents had already been put in place by various health structures. Community-level actions such as awareness raising and community engagement to improve communication were identified; furthermore, strengthening the health system with the use of security, implementing disciplinary measures in the workplace, capacity building of HCWs, strengthening collaborative relationships between staff, resolving conflicts amicably, and improving the planning of human resources and compensation have been established (55.3%) in some health facilities. These measures are consistent with studies recommending the need for strengthening the security system to reduce aggression [38, 48], increasing the penalties for various offences, strengthening disciplinary measures [63], awareness raising [48] and training of HCWs to improve their communication skills with patients [46].

### Limitations and strengths of the study

It would have been preferrable to include political and administrative authorities as well as the community in the interviewees to get their opinions on aggression against HCWs. However, we were constrained by limited resources. The fact that in the quantitative study we recruited only HCWs who were conveniently accessible and present the day of the survey does not allow us to generalise the findings to all HCWs.

Finally, due to the sensitivity of the issue in the context of security instability, some HCWs declined to have their interviews recorded. In this case, the interviewer took notes directly during the interview. Nevertheless, we are confident of the results of the study with a 98% response rate.

The strength of this study was the preparatory phase of data collection involving the health authorities (provincial health division heads) to inform HCWs of the arrival of the data collection team and the importance of this research in the 2 provinces. This was one of the major factors that enabled us to reach the greatest number of respondents despite the sensitive nature of the research topic.

## Conclusions

In a setting where ongoing armed conflicts continue to destabilize the entire healthcare sector, our study shows that HCWs are subjected to various types of violence during epidemic emergencies.

This aggression is perpetrated by patients, their families, members of the community and armed groups as well as by on-duty colleagues, demonstrating that HCWs are not protected, nor respected. There is reluctance to report aggression and sanctions are ineffective in a country where there are no legal frameworks to protect HCWs. As a result, enacting strong and appropriate regulations to protect HCWs, as well as training them in appropriate approaches to manage aggression, is critical. Public distrust of the health system is a major gap that needs to be filled in order to reduce attacks on the HCWs.

## Data Availability

The datasets used and/or analysed during the current study are available from the corresponding author on reasonable request.

## Declarations

### Disclaimer

Jessica Ramirez Mendoza confirms that the views and opinions expressed in this publication do not in any way constitute the official view or position of the ICRC. Every effort has been made to comply with my duty of discretion regarding activities undertaken during my employment/missions with the ICRC.

## Abbreviations

COZ: Central office of the health zone
DRC: Democratic Republic of Congo
GRH: General Reference Hospital
HC: Health Centers
HCWs: Health Care Workers
HF: Health Facilities
HZ: Health Zone
ICRC: International Committee of the Red Cross
NGO: Non Governmental Organization
PHD: Provincial Hospital District
OCHA: United Nations Office for the Coordination of Humanitarian Affairs
WHO: World Health Organization

## References

[1] The World Medical Association. 73rd World Health Assembly Agenda item 3: Covid-19 pandemic response. Available from: https://www.wma.net/wp-content/uploads/2020/05/WHA73-WMA-statement-on-Covid-19-pandemic-response-.pdf.

[2] International Labour Office, World Health Organization. Occupational safety and health in public health emergencies: A manual for protecting health workers and responders. World Health Organization. Geneva. 2020. Available from: https://apps.who.int/iris/bitstream/handle/10665/275385/9789241514347-eng.pdf?ua=1&ua=1

[3] Lafta R, Qusay N, Mary M, Burnham G. Violence against doctors in Iraq during the time of COVID-19. PLoS One. 2021;16(8 August):1–12. 10.1371/journal.pone.0254401. 10.1371/journal.pone.0254401 PMC8345879 34358239

[4] WHO, UNODC, UNDP. Global status report on violence prevention 2014. WHO, Geneva S. 2014 [cited 2022 Jul 30]. Available from: https://www.who.int/publications/i/item/9789241564793.

[5] Violence Prevention Alliance Approach. [cited 2022 Jul 31]. Available from: https://www.who.int/groups/violence-prevention-alliance/approach.

[6] World Health Organization. Strengthening the health system response to violence against women in Uganda: Lessons learned from adapting and implementing WHO guidelines and tools. 2020.

[7] ICRC. Geneva Conventions of 1949 and Additional Protocols, and their Commentaries. [cited 2022 Aug 19]. Available from: https://ihl-databases.icrc.org/applic/ihl/ihl.nsf/vwTreaties1949.xsp.

[8] ICRC Database. International Humanitarian Law Databases - ICRC [cited 2023 Feb 18]. Available from: https://ihl-databases.icrc.org/en.

[9] Michlig GJ, Lafta R, Al-Nuaimi M, Burnham G. Providing healthcare under ISIS: A qualitative analysis of healthcare worker experiences in Mosul, Iraq between June 2014 and June 2017. Glob Public Health. 2019 [cited 2022 Aug 21];14(10):1414–27. Available from: https://www.tandfonline.com/doi/abs/10.1080/17441692.2019.1609061. 10.1080/17441692.2019.1609061 31034779

[10] Kallström A, Al-Abdulla O, Parkki J, Häkkinen M, Juusola H, Kauhanen J. I don’t leave my people; They need me: Qualitative research of local health care professionals’ working motivations in Syria. Confl Health. 2022;16(1):1–11. 10.1186/s13031-021-00432-y. 10.1186/s13031-021-00432-y PMC8721480 34980205

[11] ICRC. ICRC institutional Health Care in Danger strategy 2020–2022 Protecting health care from violence and attacks in situations of armed conflict and other emergencies 1. 2020;1–10.

[12] ICRC. Ministers of Health Meeting on Protection of Health Care from Violence. 2022.

[13] Geneva S. ; 2020. Available from: https://healthcareindanger.org/wp-content/uploads/2020/08/4448_002_HCID_selected_experiences_2_ICRC.pdf.

[14] ICRC. Changing behaviour Tackling violence against health care in Niger, The Central African Republic and Nigeria Selected experiences. ICRC. Geneva. 2018. Available from: https://healthcareindanger.org/wp-content/uploads/2018/11/4369_002_HCID_selected_experiences_web.pdf.

[15] Muzembo BA, Mbutshu LH, Ngatu NR, Malonga KF, Eitoku M, Hirota R et al. Workplace violence towards Congolese health care workers: a survey of 436 healthcare facilities in Katanga province, Democratic Republic of Congo. J Occup Health. 2015 [cited 2022 Aug 20];57(1):69–80. Available from: https://pubmed.ncbi.nlm.nih.gov/25476862/. 10.1539/joh.14-0111-OA 25476862

[16] Insecurity Insight. Safeguarding Health in Conflict. Democratic Republic of the Congo: Violence Against Health Care in Conflict 2021 - Democratic Republic of the Congo | ReliefWeb. [cited 2022 Aug 20]. Available from: https://reliefweb.int/report/democratic-republic-congo/democratic-republic-congo-violence-against-health-care-conflict-2021.

[17] Davies S, Pettersson T, Berg, MO¨. Organized violence 1989–2021 and drone warfare: J Peace Res. 2022;59(4):593–610. Available from: https://journals.sagepub.com/doi/full/10.1177/00223433221108428.

[18] Centers for Disease Control and Prevention (CDC) (2003). Elevated mortality associated with armed conflict–democratic Republic of Congo, 2002. MMWR Morb Mortal Wkly Rep.

[19] Green A. DR Congo Ebola virus treatment centres attacked. Lancet (London, England). 2019 [cited 2023 Feb 18];393(10176):1088. Available from: http://www.thelancet.com/article/S0140673619305768/fulltext. 10.1016/S0140-6736(19)30576-8 30957744

[20] Mueller ED, Rebmann T. Analyzing Targeted Violence Against Medical Workers and EVD Incidence in the 2018-19 Democratic Republic of the Congo Outbreak Using Vector Autoregression and Granger Causality. Heal Secur. 2019 [cited 2022 Aug 20];17(6):477–84. Available from: https://pubmed.ncbi.nlm.nih.gov/31859571/. 10.1089/hs.2019.0087 31859571

[21] Juma CA, Mushabaa NK, Salam FA, Ahmadi A, Lucero-Prisno DE. COVID-19: The Current Situation in the Democratic Republic of Congo. Am J Trop Med Hyg. 2020 [cited 2022 Aug 20];103(6):2168–70. Available from: https://www.ajtmh.org/view/journals/tpmd/103/6/article-p2168.xml. 10.4269/ajtmh.20-1169 PMC7695113 33050981

[22] World bank blogs. Reversing the adverse effects of the COVID-19 pandemic in the Democratic Republic of Congo. World bank blogs. 2021 [cited 2022 Aug 20]. Available from: https://blogs.worldbank.org/africacan/reversing-adverse-effects-covid-19-pandemic-democratic-republic-congo.

[23] Programs JHC. for C. Lack of Understanding, Trust Allows Ebola to Spread in DRC. Johns Hopkins Center for Communication Programs Apr, 2019.

[24] CFR. Distrust at Core of Ebola Crisis in Eastern Congo. Council on foreign relations. 2019 [cited 2023 Feb 18]. Available from: https://www.cfr.org/blog/distrust-core-ebola-crisis-eastern-congo.

[25] Roberts N (2021). MSF and Ebola in Nord Kivu: Positioning, Politics and Pertinence. J Humanit Aff.

[26] Médecins Sans Frontières. DRC tenth Ebola outbreak. 2020 [cited 2022 Aug 20]. Available from: https://www.msf.org/drc-tenth-ebola-outbreak.

[27] Congo Research Group KST, Center on International Cooperation. The landscape of armed groups in Eastern Congo, missed opportunities. Protracted Insecurity and Self-Fulfilling Prophecies. 2021.

[28] John W, Creswell VL, Plano. Clark. Designing and Conducting Mixed Methods Research. SAGE Publi. John W. Creswell, Vicki L. Plano Clark, editors. SAGE Publications; 2017 [cited 2022 Jun 1]. 520 p. Available from: https://us.sagepub.com/en-us/nam/designing-and-conducting-mixed-methods-research/book241842.

[29] Pinard R, Potvin P, Rousseau R (2022). Le choix d’une approche méthodologique mixte de recherche en éducation. Rech Qual.

[30] Braun V, Clarke V (2006). Using thematic analysis in psychology. Qual Res Psychol.

[31] Deutsche Welle (DW). Rebels attack UN peacekeepers in DRC, with more than a dozen dead and scores wounded. 2019 [cited 2023 Feb 18]. Available from: https://www.dw.com/en/rebels-attack-un-peacekeepers-in-drc-with-more-than-a-dozen-dead-and-scores-wounded/a-41718228.

[32] UN News. Ebola-hit DRC faces “perfect storm” as uptick in violence halts WHO operation. 2018 [cited 2023 Feb 18]. Available from: https://news.un.org/en/story/2018/09/1020392.

[33] Seun-Fadipe CT, Akinsulore AA, Oginni OA. Workplace violence and risk for psychiatric morbidity among health workers in a tertiary health care setting in Nigeria: Prevalence and correlates. Psychiatry Res. 2019 [cited 2023 Feb 18];272:730–6. Available from:, Azagoh-Kouadio R, Yao KC. 10.1016/j.psychres.2018.12.177 30832193

[34] Aka-Tanoh KA, Avi C, Yeboua KR et al. Violence on Health Professionals: Experience of the Obstetrics & Gynecology and Pediatrics Departments at the University Teaching Hospital of Bouaké, Côte d’Ivoire. Open J Pediatr. 2018 [cited 2023 Feb 18];8(1):8–18. Available from: http://www.scirp.org/journal/PaperInformation.aspx?PaperID=82750.

[35] Kitaneh M, Hamdan M. Workplace violence against physicians and nurses in Palestinian public hospitals: a cross-sectional study. BMC Health Serv Res. 2012 [cited 2022 Aug 20];12(1). Available from: https://pubmed.ncbi.nlm.nih.gov/23256893/. 10.1186/1472-6963-12-469 PMC3541970 23256893

[36] Lafta RK, Falah N. Violence against health-care workers in a conflict affected city. Med Confl Surviv. 2019 [cited 2022 Aug 20];35(1):65–79. Available from: https://pubmed.ncbi.nlm.nih.gov/30406677/. 10.1080/13623699.2018.1540095 30406677

[37] Mahase E (2019). Attacks on Ebola sites in DRC leave four workers dead. BMJ.

[38] Al Anazi RB, Alqahtani SM, Mohamad AE, Hammad SM, Khleif H. Violence against Health-Care Workers in Governmental Health Facilities in Arar City, Saudi Arabia. Sci World J. 2020 [cited 2022 Aug 20];2020:6. Available from: https://pubmed.ncbi.nlm.nih.gov/32256248/. 10.1155/2020/6380281 PMC7109551 32256248

[39] Rafeea F, Al Ansari A, Abbas EM, Elmusharaf K, Abu Zeid MS. Violence toward health workers in Bahrain Defense Force Royal Medical Services’ emergency department. Open Access Emerg Med. 2017 [cited 2022 Aug 20];9:113. Available from: https://www.pmc/articles/PMC5687482/. 10.2147/OAEM.S147982 PMC5687482 29184452

[40] Abdellah RF, Salama KM. Prevalence and risk factors of workplace violence against health care workers in emergency department in Ismailia, Egypt. Pan Afr Med J. 2017 [cited 2022 Aug 20];26. Available from: https://pubmed.ncbi.nlm.nih.gov/28451000/. 10.11604/pamj.2017.26.21.10837 PMC5398248 28451000

[41] McLean D. Violence and its Humanitarian Impact: The Case of Kivu. MSF-analysis. [cited 2023 Feb 18]. Available from: https://msf-analysis.org/violence-humanitarian-impact-case-kivu/.

[42] Madzhadzhi LP, Akinsola HA, Mabunda J, Oni HT. Workplace Violence Against Nurses: Vhembe District Hospitals, South Africa. Res Theory Nurs Pract. 2017 [cited 2023 Feb 18];31(1):28–38. Available from: https://pubmed.ncbi.nlm.nih.gov/28196575/. 10.1891/1541-6577.31.1.28 28196575

[43] Kraemer MUG, Pigott DM, Hill SC, Vanderslott S, Reiner RC, Stasse S et al. Dynamics of conflict during the Ebola outbreak in the Democratic Republic of the Congo 2018–2019. BMC Med. 2020;18(1). 10.1186/s12916-020-01574-1 PMC7184697 32336281

[44] Women in Global Health. Her stories: ending sexual exploitation, abuse and harassment of women health workers. 2022. Available from: WGH-Her-Stories-SEAH-Report_Policy-Report-Dec-2022.pdf (womeningh.org).

[45] Kwanjo Banda C, Mayers P, Duma S. Violence against nurses in the southern region of Malawi. Heal SA Gesondheid. 2016 [cited 2023 Feb 18];21(0):415–21. Available from: https://hsag.co.za/index.php/hsag/article/view/1000.

[46] Kibunja BK, Musembi HM, Kimani RW, Gatimu SM. Prevalence and Effect of Workplace Violence against Emergency Nurses at a Tertiary Hospital in Kenya: A Cross-Sectional Study. Saf Health Work. 2021 [cited 2022 Aug 20];12(2):249–54. Available from: https://pubmed.ncbi.nlm.nih.gov/34178404/. 10.1016/j.shaw.2021.01.005 PMC8209348 34178404

[47] Weldehawaryat HN, Weldehawariat FG, Negash FG (2020). Prevalence of Workplace Violence and Associated factors against nurses working in Public Health Facilities in Southern Ethiopia. Risk Manag Healthc Policy.

[48] Zafar W, Siddiqui E, Ejaz K, Shehzad MU, Khan UR, Jamali S et al. Health care personnel and workplace violence in the emergency departments of a volatile metropolis: results from Karachi, Pakistan. J Emerg Med. 2013 [cited 2022 Aug 20];45(5):761–72. Available from: https://pubmed.ncbi.nlm.nih.gov/24011477/. 10.1016/j.jemermed.2013.04.049 PMC4332856 24011477

[49] Park SJ, Brown H, Wema KM, Gobat N, Borchert M, Kalubi J et al. “Ebola is a business”: an analysis of the atmosphere of mistrust in the tenth Ebola epidemic in the DRC. Crit Public Health. 2022 [cited 2023 Feb 18]; Available from: https://www.tandfonline.com/doi/abs/10.1080/09581596.2022.2128990.

[50] McLean D. Plagues of Prejudice. Cassandra Voices. 2020 [cited 2023 Feb 18]. Available from: https://cassandravoices.com/history/plagues-of-prejudice/.

[51] Earle-Richardson G, Erlach E, Walz V, Baggio O, Kurnit M, Camara CA (2021). New mixed methods Approach for Monitoring Community perceptions of Ebola and Response efforts in the Democratic Republic of the Congo. Glob Heal Sci Pract.

[52] Cai R, Tang J, Deng C, Lv G, Xu X, Sylvia S et al. Violence against health care workers in China, 2013–2016: Evidence from the national judgment documents. Hum Resour Health. 2019 [cited 2022 Aug 20];17(1):1–14. Available from: https://human-resources-health.biomedcentral.com/articles/10.1186/s12960-019-0440-y. 10.1186/s12960-019-0440-y PMC6933725 31878939

[53] Waleed M, Algwaiz, Saad A, Alghanim. Violence exposure among health care professionals in Saudi public hospitals. A preliminary investigation - PubMed. Saudi Med J. 2012 [cited 2022 Aug 20];33(1):76–82. Available from: https://pubmed.ncbi.nlm.nih.gov/22273653/. 22273653

[54] Chakraborty S, Mashreky SR, Dalal K. Violence against physicians and nurses: a systematic literature review. J Public Health (Bangkok). 2022 [cited 2022 Aug 20];30(8):1837–55. Available from: https://pubmed.ncbi.nlm.nih.gov/35096514/. 10.1007/s10389-021-01689-6 PMC8783572 35096514

[55] Aljohani B, Burkholder J, Tran QK, Chen C, Beisenova K, Pourmand A. Workplace violence in the emergency department: a systematic review and meta-analysis. Public Health. 2021 [cited 2022 Aug 20];196:186–97. Available from: https://pubmed.ncbi.nlm.nih.gov/34246105/. 10.1016/j.puhe.2021.02.009 34246105

[56] Talas MS, Kocaöz S, Akgüç S. A survey of violence against staff working in the emergency department in ankara, Turkey. Asian Nurs Res (Korean Soc Nurs Sci). 2011 [cited 2022 Aug 20];5(4):197–203. Available from: https://pubmed.ncbi.nlm.nih.gov/25030520/. 10.1016/j.anr.2011.11.001 25030520

[57] Elrha ICRC. RAND. Researching violence against health care: gaps and priorities. Elhra. Global; 2020. Available from: https://www.elrha.org/wp-content/themes/elrha/pdf/elrha-and-icrc-violence-against-health-care-full-report-010720-digital.pdf.

[58] ICRC. Colombia: health care in danger. 2022 Mar. Available from: https://www.icrc.org/en/document/colombia-health-care-danger.

[59] ALBashtawy M, Aljezawi M. Emergency nurses’ perspective of workplace violence in Jordanian hospitals: A national survey. Int Emerg Nurs. 2016 [cited 2022 Aug 20];24:61–5. Available from: https://pubmed.ncbi.nlm.nih.gov/26188629/. 10.1016/j.ienj.2015.06.005 26188629

[60] Hamdan M, Abu Hamra A. Workplace violence towards workers in the emergency departments of Palestinian hospitals: a cross-sectional study. Hum Resour Health. 2015 [cited 2022 Aug 21];13(1). Available from: https:///www.pmc/articles/PMC4435901/. 10.1186/s12960-015-0018-2 PMC4435901 25948058

[61] Al-Turki N, Afify AAM, Alateeq M. Violence against health workers in Family Medicine Centers. J Multidiscip Healthc. 2016 [cited 2022 Aug 20];9:257. Available from: /pmc/articles/PMC4898428/. 10.2147/JMDH.S105407 PMC4898428 27330300

[62] Gerberich SG, Church TR, McGovern PM, Hansen HE, Nachreiner NM, Geisser MS et al. An epidemiological study of the magnitude and consequences of work related violence: the Minnesota Nurses’ Study. Occup Environ Med. 2004 [cited 2022 Aug 20];61(6):495. Available from:/pmc/articles/PMC1763639/?report = abstract. 10.1136/oem.2003.007294 PMC1763639 15150388

[63] El-Gilany AH, El-Wehady A, Amr M. Violence against primary health care workers in Al-Hassa, Saudi Arabia. J Interpers Violence. 2010 [cited 2022 Aug 20];25(4):716–34. Available from: https://pubmed.ncbi.nlm.nih.gov/19494243/. 10.1177/0886260509334395 19494243

